# A case of perirenal extramedullary hematopoiesis in a patient with primary myelofibrosis

**DOI:** 10.1007/s13730-017-0274-1

**Published:** 2017-09-11

**Authors:** Kentaro Imai, Tomonori Aoi, Hiroki Kitai, Nobuhide Endo, Masahiko Fujino, Shizunori Ichida

**Affiliations:** 1 Department of Nephrology, Japanese Red Cross Nagoya Daiichi Hospital, 3-35 Michishita-cho, Nakamura-ku, Nagoya, Aichi Japan; 2 Department of Pathology, Japanese Red Cross Nagoya Daiichi Hospital, Nagoya, Japan

**Keywords:** Extramedullary hematopoiesis, Primary myelofibrosis, Kidney biopsy, Contrast-enhanced computer tomography

## Abstract

Extramedullary hematopoiesis (EMH) is hematopoiesis in organs outside the bone marrow and most frequently occurs in the liver, spleen, and lymph nodes. We report a case of perirenal EMH revealed by kidney biopsy in a patient with primary myelofibrosis. We observed only bilateral kidney enlargement with plain computed tomography (CT) and ultrasonography before obtaining a renal biopsy. We obtained a percutaneous biopsy from the lower pole of the left kidney using ultrasonographic guidance. Ultrasonography just after the renal biopsy revealed no bleeding around the kidney. However, early the next morning, the patient developed severe hemorrhagic shock. Contrast-enhanced CT at that time revealed a massive hematoma in the left posterior perirenal space and bilateral abnormalities of the perirenal soft tissues. In patients with primary myelofibrosis, if plain CT shows an abnormal renal enlargement, EMH should be considered. In addition, a contrast-enhanced CT should be obtained before performing a percutaneous renal biopsy to assess for the possibility of perirenal EMH in these patients.

## Introduction

Extramedullary hematopoiesis (EMH) is defined as the development and growth of hematopoietic tissue outside the bone marrow and occurs in patients with various hematologic disorders, including myelofibrosis, chronic myeloid leukemia, and polycythemia vera [1].

EMH commonly occurs in the liver and spleen, resulting in hepatosplenomegaly. EMH has also been described in other sites including the kidney [2].

Herein, we report a case of perirenal EMH in a patient with primary myelofibrosis that was diagnosed with the occurrence of massive bleeding after a percutaneous renal biopsy.

## Case report

A 58-year-old man who had been diagnosed with polycythemia vera 13 years previously developed myelofibrosis. He underwent bone marrow transplantation 8 years previously, followed 1 year later by a recurrence of polycythemia vera for which he was treated with hydroxycarbamide. Since that time, his serum creatinine level had gradually increased to 1.59 mg/dL at the time of presentation. The urine occult blood test was negative, but a random urine protein-to-creatinine ratio was 1.0 g/gCr. He was then referred to our hospital for renal biopsy to determine the etiology of his renal insufficiency, including possible bone marrow transplant nephropathy or drug-induced interstitial nephritis.

On admission, the patient was 172 cm tall, his body weight was 62 kg, and he was alert and communicative. His blood pressure was 119/72 mmHg. Physical examination showed hepatosplenomegaly, but no other abnormal signs were noted. He had no family history of bleeding diathesis and no exposure to medications associated with hemorrhagic complications.

**Fig. 1:**
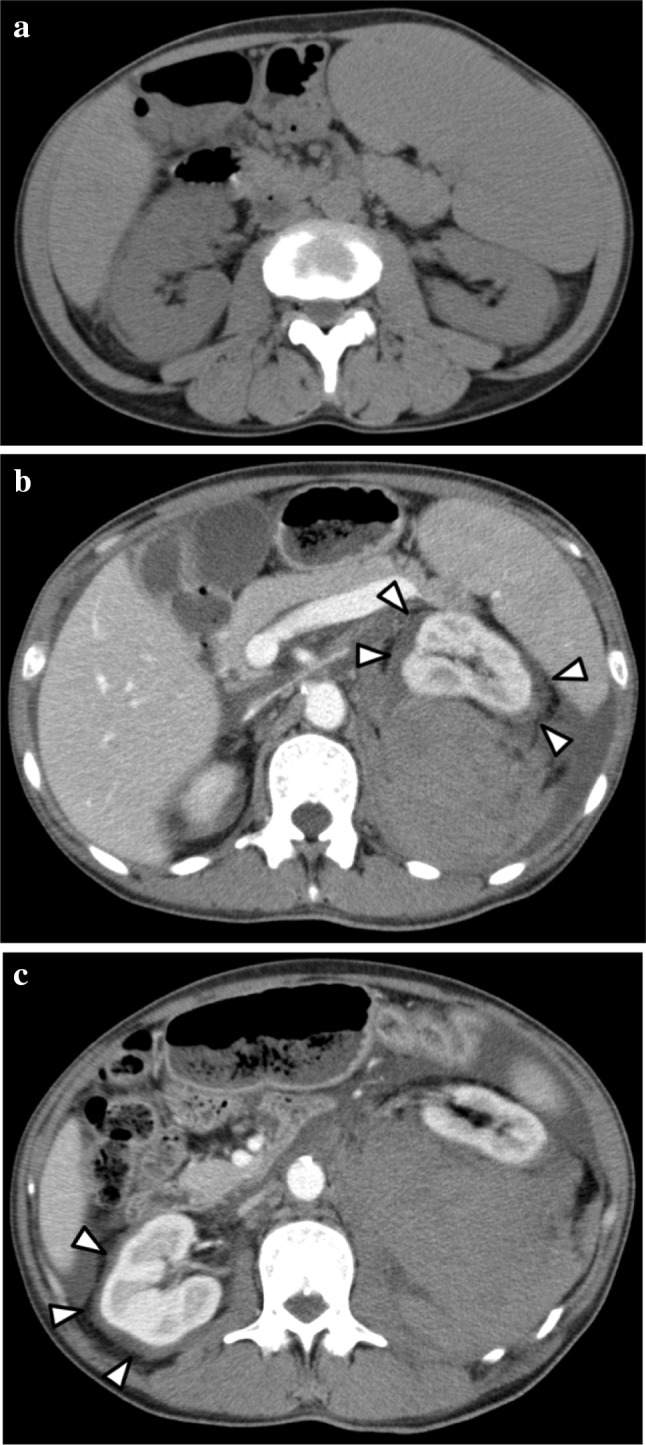
**a** Plain computer tomography before renal biopsy shows only severe hepatosplenomegaly and bilateral kidney enlargement. **b**, **c** Contrast-enhanced computer tomography after renal biopsy shows a massive hematoma in the left posterior perirenal space and non-enhancing tissue with a thickness of 8 mm around both kidneys

Laboratory test results revealed impaired renal function with a serum creatinine level of 1.29 mg/dL and an estimated glomerular filtration rate (eGFR) of 45.8 mL/min/1.73 m^2^. The hemoglobin level was 10.0 g/dL, and the platelet count was 38.3 × 10^4^/mm^3^. Coagulation studies showed a prothrombin ratio (PT-INR) of 1.23 and an activated partial thromboplastin time (APTT) of 37.8 s. The patient’s laboratory data are summarized in Table 1. A plain computer tomography (CT) scan revealed bilateral kidney enlargement (Fig. 1a).

**Table 1:** Laboratory data on admission

Biochemical test		Complete blood test		Immunological test	
CRP	0.06 mg/dL	WBC	19200/mm^3^	IgG	2879 mg/dL
TP	8.7 g/dL	Neutrophil	67.1%	IgA	844 mg/dL
Alb	3.7 g/dL	Lymphocyte	15.3%	IgM	65 mg/dL
T-bili	0.5 mg/dL	Eosinophil	3.8%	C3	89 mg/dL
AST	42 IU/L	Monocyte	10.8%	C4	28 mg/dL
ALT	25 IU/L	RBC	320 × 10^4^/mm^3^	CH50	44.8CH50/mL
LDH	903 IU/L	Hb	10.0 g/dL	ANA	(–)
ALP	548 IU/L	Ht	31.7%	MPO-ANCA	<1.0 EU
CK	187 IU/L	Ret	2.28%	IgG4	286 mg/dL
Na	136 mEq/L	MCV	110.9 fL	Electrophoresis	M protein (–)
K	4.6 mEq/L	PLT	38.3 × 10^4^/mm^3^	Serum protein fraction	
Cl	103 mEq/L	Urinalysis		Alb	47.5%
Ca	8.1 mg/dL	Protein	2.6 g/gCr	α1	2.7%
P	4.1 mg/dL	Occult	(–)	α2	5.3%
BUN	23 mg/dL	RBC	<1/HPF	β	7.7%
UA	7.8 mg/dL	Cast	<1/HPF	γ	36.8%
Cre	1.29 mg/dL	β2MG	25168 μg/L		
T-chol	118 mg/dL	Electrophoresis	BJP (–)		
HbA1c	5.4%				

*CRP* C reactive protein, *TP* total protein, *Alb* albumin, *T-bili* total bilirubin, *AST* aspartate aminotransferase, *ALT* alanine aminotransferase, *LDH* lactate dehydrogenase, *ALP* alkaline phosphatase, *CK* creatine phosphokinase, *BUN* blood urea nitrogen, *UA* urinalysis, *Cre* creatinine, *T-chol* total cholesterol, *HbA1c* hemoglobin A1c, *Hb* hemoglobin, *Ht* hematocrit, *Ret* reticulocyte count, *MCV* mean corpuscular volume, *HPF* high power field, *BJP* Bence-Jones protein, *ANA* antinuclear antibody, *MPO-ANCA* myeloperoxidase-anti-neutrophil cytoplasmic antibody

On day 2, a percutaneous biopsy of the lower pole of the left kidney was performed using ultrasonographic guidance. Ultrasonography immediately after the biopsy showed no bleeding around the kidney, and his vital signs were stable. Early the next morning, the patient was found to have a blood pressure of 67/45 mmHg and a contrast-enhanced CT scan was ordered. The scan revealed a massive hematoma in the left posterior perirenal space and non-enhancement of the tissues surrounding both kidneys (Fig. 1b, c). Fortunately, leakage of contrast medium from the kidney was not observed, and his vital signs stabilized with the administration of blood transfusions alone.

**Fig. 2:**
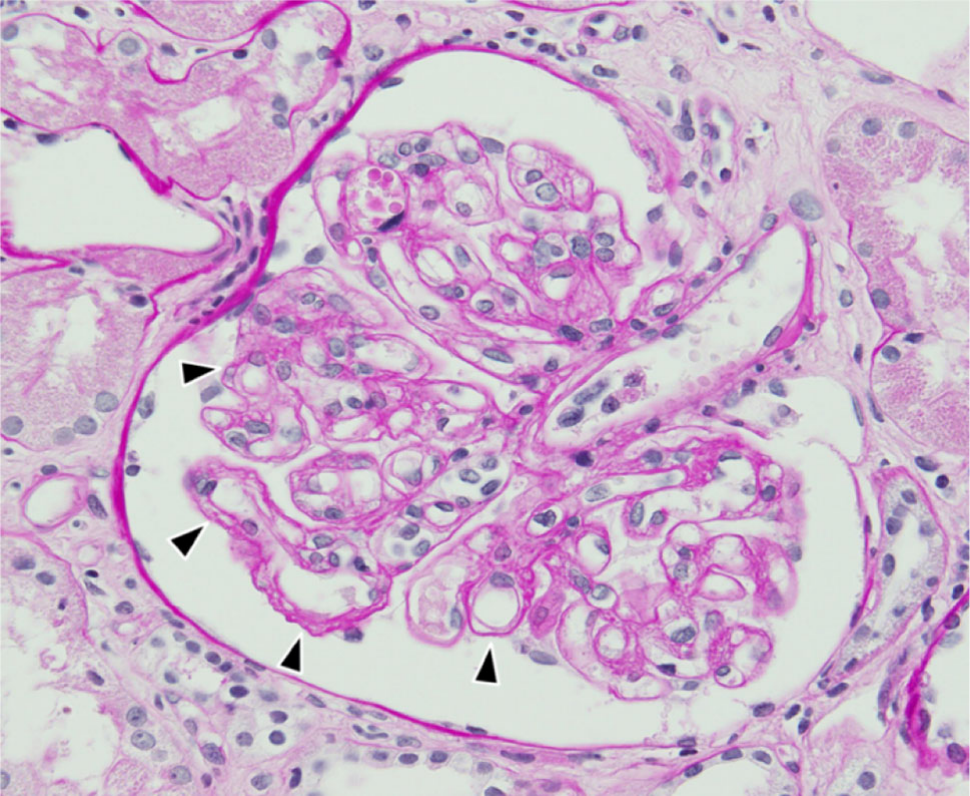
Photomicrograph of renal biopsy specimen shows endothelial swelling and widening of the subendothelial space, suggesting endothelial cell damage (periodic acid–Schiff stain ×400)

The biopsy specimen revealed endothelial swelling and widening of the subendothelial space, suggesting endothelial cell damage (Fig. 2), possibly caused by bone marrow transplantation or medications such as calcineurin inhibitors. No glomeruli were seen on immunofluorescent and electronic microscope studies.

**Fig. 3:**
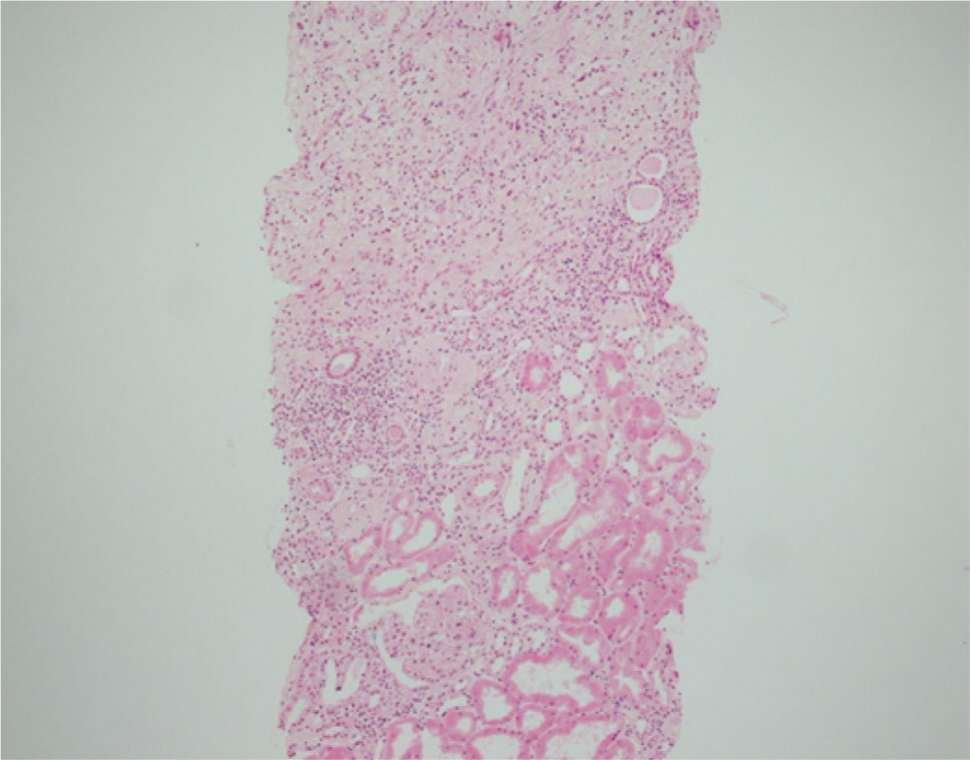
Photomicrograph of renal biopsy showing edematous fibrous tissue outside the renal capsule and extending into the renal parenchyma. The kidney capsule is not clearly identified (hematoxylin and eosin ×40)

**Fig. 4:**
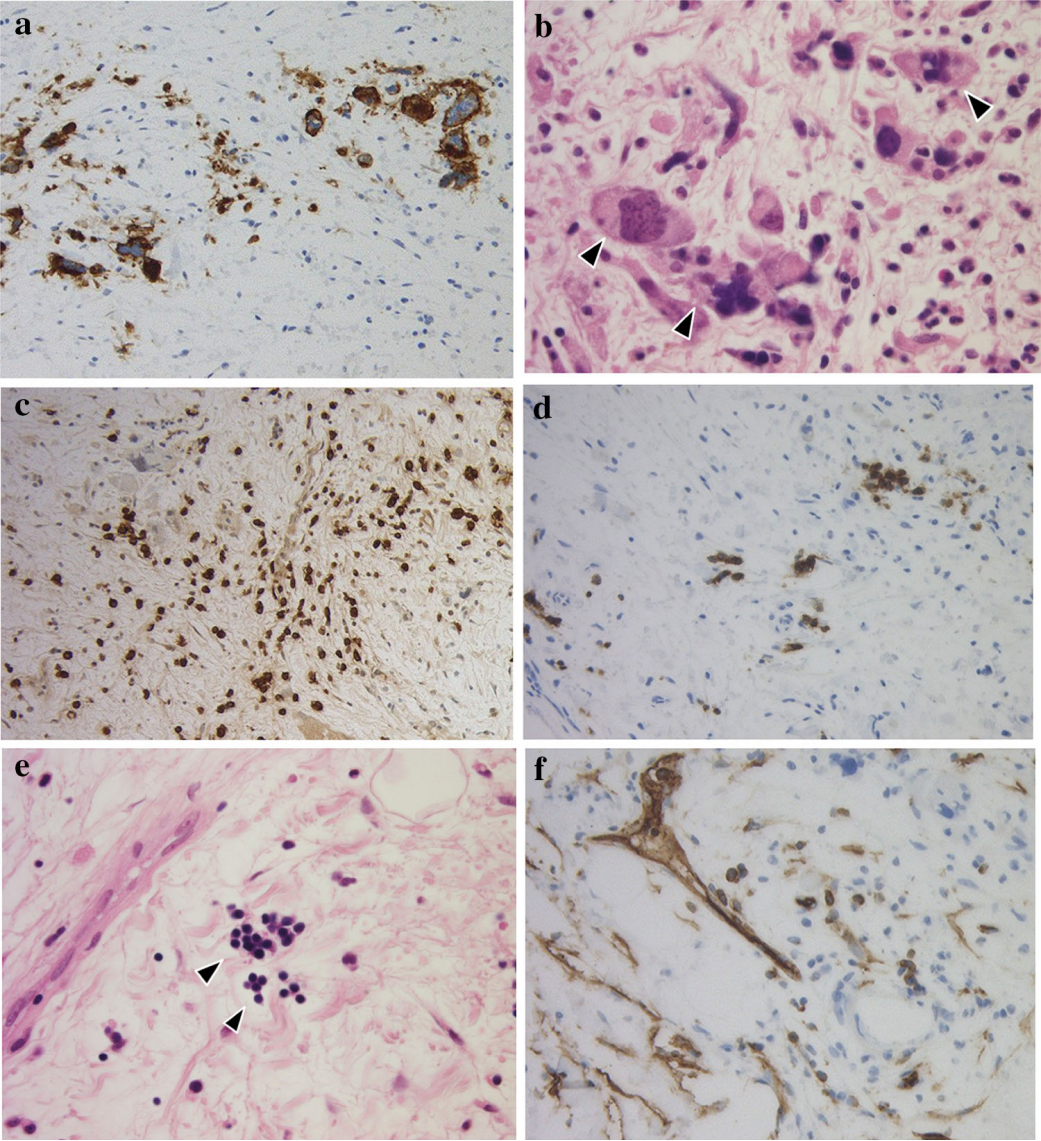
Immunohistochemical staining revealed **a**, **b** CD42b-positive megakaryocytes, **c** MPO-positive granulocytes/monocytes, **d**, **e** CD71-positive erythroblasts, and **f** CD34-positive blasts in the edematous fibrous tissue outside the kidney

Histopathology also revealed edematous fibrous tissue outside the kidney capsule partially extending into the renal parenchyma (Fig. 3). Immunohistochemical staining revealed CD42b-positive megakaryocytes, MPO-positive granulocyte/monocytes, CD71-positive erythroblasts, and CD34-positive blasts in these tissues (Fig. 4). A diagnosis of extramedullary hematopoiesis associated with myelofibrosis was made.

The histological findings of the tissues outside the kidney capsule and in the renal parenchyma were essentially the same. Reduced renal function was thought to be mainly due to a combination of endothelial cell damage caused by post-transplant or medication-induced nephropathy and invasion of extramedullary hematopoietic tissue into the kidney parenchyma, and we chose to continue treatment of the primary disease. The tissue outside the kidney capsule was rich in blood vessels and appeared to be hemorrhagic, resulting in major bleeding after the renal biopsy.

## Discussion

Myeloproliferative neoplasms (MPNs) are clonal hematopoietic stem cell disorders defined by expansion of one or more myeloid cell lines. MPN occurs mainly in older adults [3].

Primary myelofibrosis is characterized by bone marrow fibrosis and the proliferation of megakaryocytes and granulocytes in the bone marrow, and may result in progressive pancytopenia and EMH.

EMH is defined as the growth and development of hematopoietic tissue outside the bone marrow and is seen in patients with several hematologic disorders, including myelofibrosis, chronic myeloid leukemia, essential thrombocythemia, sickle-cell anemia, and polycythemia vera.

Although the most frequent site of EMH is the reticuloendothelial system, including the liver, spleen, and lymph nodes, it may be found in other sites. EMH can be widespread in patients with advanced myelofibrosis, especially after splenectomy [4]. Several other organs have been reported to be involved by EMH, including the middle ear, pancreas, pharynx, lung, pleura, heart, pericardium, gastrointestinal tract, peritoneum, thyroid, and skin. Renal EMH is uncommon but occurs most often as intraparenchymal lesions and has also been described in renal allografts [5].

Alexander et al. reported 14 patients with renal EMH on kidney biopsy specimens from 1994 to 2015 [6]. All presented with reduced renal function, including five (36%) patients with acute renal failure. The mean serum creatinine at the time of renal biopsy was 2.9 mg/dL (range 1.2–7.3 mg/dL). All had proteinuria (mean 7.9 g/24 h; range 0.5–28 g/24 h), including nine patients with urine protein levels ≥3 g/24 h. Renal EMH appeared histologically as an interstitial infiltration (*n* = 12) and/or a perirenal infiltration (*n* = 3) or mass-like lesion (*n* = 1). Previously reported cases of patients with perirenal EMH are summarized in Table 2.

**Table 2:** Reported cases of perirenal extramedullary hematopoiesis

Patient	References	Age/gender	Renal presentation	Hematological disorder	Other sites of EMH	Serum creatinine at presentation (mg/dL)	Proteinuria (g/24 h)	Treatment
1	Cheng et al. [10]	69/F	Chronic kidney disease	Primary myelofibrosis	NA	6.7	NA	Peritoneal dialysis Janus kinase inhibitor
2	Alexander et al. [6]	87/M	Acute renal failure and proteinuria	Primary myelofibrosis	None	2.3	3.2	Maintained on pomalidomide
3	Alexander et al. [6]	72/M	Renal failure and proteinuria	Myeloproliferative neoplasm, not otherwise specified	Spleen	3.4	3.7	Steroids
4	Alexander et al. [6]	65/F	Acute renal failure and proteinuria	Primary myelofibrosis	Liver and spleen	7.3	NA	NA
5	Alexander et al. [6]	67/M	Acute renal failure and proteinuria	Essential thrombocythemia	Spleen	2.5	7	Hydroxyurea, anagrelide
6	Wasylkowski et al. [12]	75/M	NA	Myelofibrosis due to polycythemia vera	Intervertebral space, pre-sacral zone, mediastinum	NA	NA	Hydroxyurea, splenectomy
7	Khandelwal et al. [9]	67/M	NA	Primary myelofibrosis		NA	NA	Splenectomy
8	Kreuziger et al. [4]	65/M	NA	Primary myelofibrosis	Liver, lymph nodes, heart, kidneys, adrenals, meninges, and pituitary gland	NA	NA	Thalidomide, splenectomy
9	Ablett et al. [13]	64/M	NA	Primary myelofibrosis	NA	NA	NA	Splenectomy
10	Choi et al. [8]	66/M	NA	Myelofibrosis with myeloid metaplasia	NA	NA	NA	Prednisone, thalidomide, cyclosporin, autologous stem cell transplantation

*NA* not available

The interstitial lesions of EMH are often confused with those of tubulointerstitial nephritis.

In addition to the interstitial lesions of EMH or interstitial nephritis, our patient’s biopsy specimen revealed endothelial cell damage due to post-transplant or medication-induced nephropathy, which is considered to be the primary cause of the patient’s reduced renal function and proteinuria.

It is important to consider the diagnosis of EMH prior to renal biopsy. In our case, though plain CT before renal biopsy showed bilateral kidney enlargement, in fact, it was considered because of perirenal EMH. In general, EMH is considered to be rich in blood vessels and hemorrhagic in nature. In our patient, there were no blood vessels large enough to cause massive bleeding seen in the patient’s biopsy specimen. EMH was believed to be the main cause of bleeding.

Scintigraphy with ^99m^Tc or ^111^InCl_3_ has been reported to be useful for establishing a diagnosis of EMH [7]. Bone marrow comprises myelopoietic, erythropoietic, megakaryopoietic, and reticuloendothelial elements. ^99m^Tc-sulfur colloid is taken up by reticuloendothelial cells in bone marrow and is used as a reticuloendothelial system imaging agent. ^111^InCl_3_ was initially developed as an agent to image only the erythropoietic component in place of iron, but reticuloendothelial cells also take up ^111^InCl_3_ when transferrin is saturated. It has been reported that few reticuloendothelial cells are present in cell blocks of specimens from soft-tissue infiltration of the renal pelvis obtained by needle aspiration biopsy. This might explain the inability of ^99m^Tc-sulfur colloid bone marrow imaging to detect renal EMH [8]. Therefore, even if ^99m^Tc-sulfur colloid bone marrow imaging is negative, EMH cannot be ruled out.

To confirm the presence of EMH, it is preferable to perform contrast-enhanced CT rather than scintigraphy. Several cases have been reported in which contrast-enhanced CT revealed perirenal EMH. Contrast-enhanced CT of the abdomen in the previous studies identified homogenous, hypodense non-enhancing rind-like soft-tissue masses in the perinephric spaces as observed in our case [4, 9]. In a previous case, MRI was performed in a patient with perirenal EMH [10], and areas of EMH show a T1-low and a T2-high signal [11]. EMH, as a cause of abnormal perirenal shadows, can be confirmed with MRI.

In conclusion, if patients with hematologic disorders, such as myelofibrosis, have kidney enlargement, the possible presence of perirenal EMH must be considered.

In these patients, we recommend contrast-enhanced CT before performing a renal biopsy because of the risk of major bleeding following the puncture of EMH tissues.
